# ClassyFlu: Classification of Influenza A Viruses with Discriminatively Trained Profile-HMMs

**DOI:** 10.1371/journal.pone.0084558

**Published:** 2014-01-03

**Authors:** Sandra Van der Auwera, Ingo Bulla, Mario Ziller, Anne Pohlmann, Timm Harder, Mario Stanke

**Affiliations:** 1 Institute for Mathematics and Computer Science, Ernst-Moritz-Arndt Universität Greifswald, Greifswald, Germany; 2 Federal Research Institute for Animal Health, Island of Riems, Greifswald, Germany; 3 Theoretical Biology and Biophysics, Group T-6, Los Alamos National Laboratory, Los Alamos, New Mexico, United States of America; Thomas Jefferson University, United States of America

## Abstract

Accurate and rapid characterization of influenza A virus (IAV) hemagglutinin (HA) and neuraminidase (NA) sequences with respect to subtype and clade is at the basis of extended diagnostic services and implicit to molecular epidemiologic studies. ClassyFlu is a new tool and web service for the classification of IAV sequences of the HA and NA gene into subtypes and phylogenetic clades using discriminatively trained profile hidden Markov models (HMMs), one for each subtype or clade. ClassyFlu merely requires as input unaligned, full-length or partial HA or NA DNA sequences. It enables rapid and highly accurate assignment of HA sequences to subtypes H1–H17 but particularly focusses on the finer grained assignment of sequences of highly pathogenic avian influenza viruses of subtype H5N1 according to the cladistics proposed by the H5N1 Evolution Working Group. NA sequences are classified into subtypes N1–N10. ClassyFlu was compared to semiautomatic classification approaches using BLAST and phylogenetics and additionally for H5 sequences to the new “Highly Pathogenic H5N1 Clade Classification Tool” (IRD-CT) proposed by the Influenza Research Database. Our results show that both web tools (ClassyFlu and IRD-CT), although based on different methods, are nearly equivalent in performance and both are more accurate and faster than semiautomatic classification. A retraining of ClassyFlu to altered cladistics as well as an extension of ClassyFlu to other IAV genome segments or fragments thereof is undemanding. This is exemplified by unambiguous assignment to a distinct cluster within subtype H7 of sequences of H7N9 viruses which emerged in China early in 2013 and caused more than 130 human infections. http://bioinf.uni-greifswald.de/ClassyFlu is a free web service. For local execution, the ClassyFlu source code in PERL is freely available.

## Introduction

Influenza A viruses (IAV) continue to threaten public as well as animal health [4], [5]. Costly diagnostic programs and in depth molecular epidemiological studies are conducted to follow the spread and to analyse evolutionary trends of avian and mammalian IAVs. Subtyping and finer phylogenetic clustering of IAVs is based on the antigenic and genetic characteristics of the immunodominant viral HA (hemagglutinin) and NA (neuraminidase) proteins and genes, respectively, into currently at least 17 HA and 10 NA subtypes [2]. Reassortment events and rapid mutation of the HA gene driven by immune-selective pressure causes antigenic shift and drift, respectively, of affected viruses [6], [7]. In addition, these processes lead to swiftly progressing phylogenetic diversification which challenges subtype and cluster assignment required for molecular epidemiological analyses [8]. Such problems culminated with the unprecedented spread of highly pathogenic avian influenza viruses (HPAIV) of subtype H5N1 within and from Asia since 2003. Tools for the reliable classification of the specific clusters are essential to follow evolutionary trends and spreading routes. As a first step towards classification of HPAIV H5N1 viruses, the H5N1 Evolution Working Group of the WHO set up a unified nomenclature system based on genetic similarity among all available HA H5(N1) sequences [1]. This annotation provides the opportunity to separate HPAIV H5N1 into different clades and lineages. Recently, the group distinguished 32 clades which are arranged as a hierarchic tree based on genetic similarities [1]. Unfortunately, there is no standard rapid method to assign newly established and uncategorised HA sequences into this system. Common approaches to assign new sequences while circumventing time-consuming phylogenetic operations include a simple BLAST [9] search against a selection of categorized HA sequences or the de novo construction of a phylogenetic tree of smaller scale of unclassified and assigned sequences. However, these methods are inhomogeneous and inconvenient since there is neither a rule how to choose the reference database for BLAST or the phylogenetic tree nor a consistent criterion to assign sequences to a specific subtype or clade. Recently, the Influenza Research Database (IRD, http://www.fludb.org) proposed the “Highly Pathogenic H5N1 Clade Classification Tool” (CT) as a free web service. IRD-CT is based on a phylogeny but keeps the tree of already classified sequences fixed [14]. Independent of the IRD development we set out to follow a different approach and assembled a tool, ClassyFlu, that is based on discriminatively trained profile hidden Markov models. Here the assignment power of ClassyFlu is examined and compared to IRD-CT and other classification approaches. We show that ClassyFlu rapidly assigns HA and NA sequences to the corresponding subtypes, and, within the H5N1 clade system, also places H5 sequences correctly, similar to IRD-CT. Moreover, we examined the influence of sequence length on the precision of clade assignment. We conclude that a sequence stretch of only 100 nucleotides spanning the HA cleavage site (so-called PanHA fragment [10]) and a stretch of 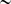
612 nucleotides (PanNA fragment [10]) at the 5′-end of the NA gene are sufficient for confident subtype assignment. However, for reliable H5N1 clade differentiation longer fragments are required.

## Materials and Methods

### HA subtypes and clades

The parameter training of our classification method requires the definition of classes to which the later input sequences shall be assigned to. While this could in principle be done de novo through sequence clustering or using phylogenetic analyses, we here used sequences and their pre-defined classification from the NCBI Influenza Virus Resource http://www.ncbi.nlm.nih.gov/genomes/FLUhttp://www.ncbi.nlm.nih.gov/genomes/FLU. In total, 63 classes comprising subtypes H1–H17 and including 32 HPAIV H5N1 clades were used. Sequences belonging to H5N1 but not to HPAIV were labeled H5N1-HPAIV and sequences belonging to H5 but not to H5N1 with H5-N1. H7 was subdivided into clusters based on a targeted selection of Eurasian and American H7 full-length HA sequences available from the EpiFlu database. We propose a clustering into 13 distinguishable clades on the basis of a phylogenetic analysis by PhyML of the HA1 encoding fragment of the hemagglutinin gene (Figure S1). A subclade was assigned if a monophyletic group of sequences was separated by aLRT [11] values 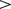
0.9 and its members distinguished on average by 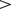
1% differences at the nucleotide level from other clusters.

The training data comprises all HA gene sequences which had a length of at least 1,600 bp and which had been released until December 2010. For the H7 subselection also recent sequences present in the Epiflu database including those of the recently emerged zoonotic H7N9 viruses from China were added. The subtype H17 is also an exception because it was described after 2010. Therefore, the only two existing sequences of H17 from 2011 were taken as training sequences and no evaluation could be performed on them. Overall, there were 15.672 sequences used for training of our classifier – 3.295 for the H5 subtype, 995 for H7 and 11.382 for all other subtypes.

### NA subtypes and clades

We selected a training set on the basis of all available non-redundant NA sequences published in GenBank until September 2013. Incomplete sequences and sequences that contain ambiguous nucleotides were excluded. Sequences possibly misclassified in GenBank were identified by a comparison to our NA reference data set. Multiple alignments of the NA sequences of each subtype were created using MAFFT [12]. A representative subset for each NA subtype N1–N9 was selected on the basis of sequence similarity. The subsets contain at least 26 and at most 109 sequences. All available three N10 sequences were selected as well.

As test set we used sequences from recent isolates published in [13]. These sequences had been characterised phylogenetically into subtypes N1–N9. We removed three of the 101 sequences of [13] that were already in our training set (KF259614, KF259642, KF259671) to obtain a test set of 98 sequences. No sequence appeared identically in both the training and test set.

### Generative training

ClassyFlu uses a specific profile hidden Markov model (HMM) for each of the 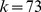 classes. For each class a multiple sequence alignment (MSA) of all sequences of that class was constucted with MUSCLE [14]. Each HMM represents the family of DNA sequences belonging to that class, including the information on conserved nucleotide positions and on position-specific probabilities of substitutions, insertions and deletions. The HMMs were built with hmmbuild from the HMMer package [15]. In doing so, the parameters of each HMM depend only on the sequences from that class. As they specify a probability distribution on DNA sequences that is fit to sequences of this class, this model is referred to as *generative*. The resulting set of 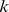 HMMs will be referred to as *database* of HMMs.

### Classification

To classify an input DNA sequence 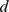 into one of the 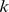 classes 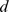 is compared to each of the 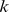 HMMs. This is done using the program hmmscan from the HMMer package. HMMer determines a log-odds score for each HMM, the logarithm of the probability of 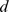 in the HMM over the probability of 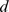 in a background model. ClassyFlu then predicts the class of an input DNA sequence 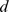 to be the HMM against which 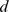 obtains the highest score. We introduced a rough filter to alert users about sequences that cannot be reliably classified. If an input sequence is long enough to be near full-length (1600 bp for HA and 1300 bp for NA) but the score is below a threshold (1600 for HA and 1300 for NA), then the classification is flagged as *not confident*.

### Discriminative training

As further described below in the evaluation section, this generatively trained database of HMM has a performance disadvantage in our setting with given classes. Although it appears to work well for the distinction between different H and N subtypes, the performance on the more difficult task of distinguishing between closer clades could be increased by our novel algorithm that aims at better discrimination and improved performance on the HPAIV H5N1 clades.

As influenza alignments contain relatively few indels, almost all parameters of the HMMs that are important for discrimination are position-specific scores for the four bases. Let 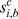 be the score of base 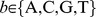 at MSA position 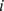 in class 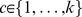. Let 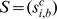 denote this set of parameters that essentially represents the HMM database. The starting point 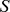 for our algorithm that improves the HMM database is taken from above *generative* database of HMMs. The supervised training algorithm outlined below iteratively makes small changes to 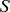. Parameter changes to 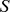 are made whenever the classification algorithm of ClassyFlu misclassifies a sequence 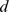 based on the current 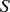. In such a case the score parameters of the two involved classes – the true class 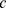 and the falsely predicted class 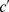 – are changed, in such a way that the score of 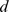 against the true HMM 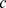 increases and its score against the false HMM 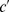 decreases. The algorithm uses a parameter 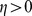 that determines how quickly the parameters of the two HMMs 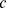 and 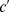 change. 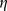 depends on the length of the input sequence 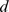. Further details of the training algorithm are described in the document ClassyFlu-algorithm.pdf that is available on the ClassyFlu web page.

### Discriminative HMM database training algorithm

**for** each DNA sequence 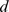 from the training set **do**

let 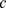 be the true class of 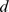


let 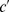 be the predicted class of 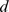 using the current HMM database parameters *S*

**if** 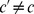 **then**

**for** all positions 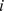 of 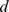 **do**

let 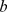 be the base in 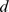 at position 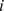


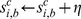


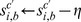



**end for**

**end if**

**end for**

The source code for this discriminative HMM-training algorithm that builds on the HMMer package is available at http://bioinf.uni-greifswald.de/ClassyFlu/query/downloads). This training prodecure is *discriminative* as it tries to *distinguish* between the given classes, rather than to model each class independently by itself. Using this algorithm, the parameters of a HMM that represents one class 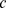 depends also on the training sequences from other classes 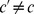. Above algorithm was used to improve only the HMMs for the H5N1 HA sequences as the performance on the other classes was good already with the generative training. The resulting database 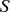 was used in the experiments in the results section as well as for the ClassyFlu web server.

## Results

### Evaluation on classified data

After the HMM database was created, the classification accuracy was determined on the subtype level and on the level of H5N1 clades.

#### HA subtype

To examine the correct assignment of a sequence to its HA subtype, test sequences of subtypes H1–H4,H6–H16, that were not included in the training were chosen from the NCBI database (Oct. 02, 2012). For this selection all available sequences with a release date starting from January 2011 were taken as input and up to 100 sequences of each subtype were chosen arbitrarily. The final set of 870 test sequences was then assigned to the specific H-subtypes. The validation resulted in a 100% accordance with respect to the reference classification provided by NCBI demonstrating the robustness of HMMs for this special task.

#### HPAIV H5N1 clades

We tested wether the finer distinction between the 32 HPAIV H5N1 clades could also be achieved in a leave-one-out cross-validation with 2,161 annotated training sequences. These sequences were taken from the NCBI database, too, while the reference annotation was adopted from the WHO nomenclature. In a first test, using the generative database of HMMs created by HMMER, 3% of the sequences were misclassified; the majority of these were classified into a neighbouring clade of the one to which it had been originally assigned. Using the discriminative training algorithm, classification performance increased from 97% to 99.2%. These measures are based on cross-validation, whereby the sequences for measuring accuracy were not used for training. The 0.8% remaining error rate corresponds to 7 disagreements out of 870 sequences. Of these, 4 sequences were assigned by ClassyFlu to the same top-level clade as in the NCBI annotation, suggesting only a minor classification error. The 3 remaining cases are the following. A/chicken/Jilin/hp/2003 assigned by ClassyFlu to clade 0 but by the WHO to clade 5. This may be due to a disputable choice in the WHO nomenclature as this sequence is the only exception to an otherwise monophyletic clade 5. A/chicken/Hong Kong/61.9/2002 and A/chicken/Hong Kong/86.3/2002 were both assigned to clade 9 by ClassyFlu but to clade 8 in the WHO nomenclature. However, these two sequences are descendants of the most recent common ancestor of all clade 9 sequences in the HA full tree http://www.who.int/entity/influenza/gisrs_laboratory/201101_h5fulltree.pdf http://www.who.int/entity/influenza/gisrs_laboratory/201101_h5fulltree.pdf also suggesting a disputable choice in the WHO classification.

#### NA subtype

All of the 98 NA full length test sequences were classified by ClassyFlu into the same subtype as they had been classified previously [13]. This perfect classification performance did not not decrease when we cut out the panNA sequence segment from each of the full length sequences and used only this smaller segment (averaging 612 bp) from the NA gene for classification. All panNA fragments were classified the same as the full length NA sequences they were extracted from.

### Application to non annotated sequences

Based on these improved classification capacities, we applied ClassyFlu to new unannotated test sequences of subtype H5 but not necessarily HPAIV H5N1. This set included all available HA genes, full DNA sequences or fragments, from the NCBI and GISAID EpiFlu influenza databases http://www.gisaid.orghttp://www.gisaid.org with a release date between and including January 2011 and September 2012 (1,033 sequences, 179 from GISAID). The classification results of ClassyFlu were compared to semiautomatic approaches using BLAST and phylogenetic analyses, and to the IRD-CT tool. For BLAST as well as for the phylogeny 196 annotated reference sequences from a multiple alignment, available from the updated WHO HPAIV nomenclature 2011, were specified (http://www.who.int/influenza/gisrs_laboratory/Smalltreeupdated.txthttp://www.who.int/influenza/gisrs_laboratory/Smalltreeupdated.txt). These sequences were used to build a local BLAST database. The subsequent evaluation with BLASTN [9] only considered results with at least 95% identity and assigned the hit with the maximum score. The same 196 sequences were used for the phylogenetic analysis (PHYLO). A subset of the non-annotated data was aligned to them using MAFFT [12] in order to build a joint phylogenetic minimum evolution tree with MEGA5 [16] which then was analysed manually for subtype and clade assignment, respectively. To achieve consistency, the method of choice was to assign the test sequences to the same clade as its nearest reference sequence in the tree.

### Classification of H5 full length HA sequences

Out of all 1,033 non-annotated test sequences extracted from NCBI and GISAID only 963 were suitable for classification with all four methods (ClassyFlu, BLAST, PHYLO, IRD-CT). The other 70 sequences were excluded since they were either too short or not sufficiently overlapping in the alignments for being classified in our phylogenetic approach (PHYLO). The evaluation of the classifications is summarized in Table 1.

**Table 1:** Pairwise comparison of the H5 HA classification of full sequences.

	ClassyFlu	BLAST	IRD-CT	PHYLO	average
ClassyFlu	–	90.8%	96.8%	98.8%	95.5%
BLAST	90.8%	-	87.1%	83.9%	89.5%
IRD-CT	96.8%	87.1%	–	89.7%	93.6%
PHYLO	98.8%	83.9%	89.7%	–	95%

Identical classification across all four methods was achieved for 87.1% of the sequences. BLAST more frequently disagreed with the other methods. ClassyFlu, IRD-CT and PHYLO proved to be very consistent as shown by the average percentage accordance in Table 1. The closest consistency in the classification is for ClassyFlu and PHYLO with 98.8% but also ClassyFlu and IRD-CT share a high value of 96.8% indicating that ClassyFlu produces results which are similarly reliable on subtype classification as those from IRD-CT and PHYLO.

### Subtype and H5 clade classification using truncated HA sequences

If correct, the classification results of sequences should be identical no matter which fragment of the input sequence is chosen. In practice, the classification accuracy increases with fragment length and sequence variability. Given the fact that variability is not uniformly distributed across the HA gene [17] it is, however, easily conceivable that the region of a fragment used for classification will influence classification accuracy. In routine diagnostics, especially of animal influenza A virus infections, shorter sequences are preferred for analysis as these can be more reliably amplified directly from clinical samples and will enable subtyping even if no culture-grown isolate was obtained. Along these lines a conventional RT-PCR method (referred to as PanHA) has recently been proposed which amplifies, independently of the subtype (H1–H16), a short 140–150 bp fragment of the HA gene that spans the endoproteolytical cleavage site of the HA protein [10]. Usable PanHA fragment sequences (without primer sequences) are 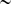 100 bp in length, i.e., less than 6% of the full length HA gene. Sequence analysis of this amplicon allows for molecular pathotyping of H5 and H7. We here examined whether the fragment is too short for reliable subtyping, and, especially, H5N1 clade assignment. Out of the 963 sequences selected for testing, 902 contained the PanHA fragment and were analysed comparatively by the four classification methods (results shown in Table 2). Not unexpectedly, classification accuracy suffered and assignments were more inconsistent: No two methods had a higher accordance than 86.7% (ClassyFlu: IRD-CT) while the minimum accordance was 39.6% (BLAST: IRD-CT).

**Table 2:** Pairwise comparison of the H5 HA classification of PanHA fragments.

	ClassyFlu	BLAST	IRD	PHYLO	average
ClassyFlu	–	42.1%	86.7%	66.7%	65.2%
BLAST	42.1%	–	39.6%	52.4%	44.7%
IRD	86.7%	39.6%	–	65.6%	64%
PHYLO	66.7%	52.4%	65.6%	–	61.6%

It should be mentioned that the IRD points to the fact that an input sequence needs to be at least 300 bp in length to ensure reliable classification with IRD-CT and that its results on shorter sequences must be treated with caution [3].

To further assess classification reliability, for each full length HA sequence the truncated HA sequence was also classified with the same tool and the classifications were compared. In the ideal case any method should classify all sequence pairs (full, PanHA) identically. The consistency was evaluated for each method individually, defined through their percentage accordance:

- IRD-CT: 87%
- ClassyFlu: 86.3%
- PHYLO: 63.5%
- BLAST: 47.1%

The outcome shows that IRD-CT and ClassyFlu achieved by far the most constant performance with 87% and 86.3% accordance, respectively. Thus, only about 13% of the H5 HA sequences differed in their clade assignment when full length sequences or the PanHA fragment were used.

As ClassyFlu and IRD-CT use quite different approaches we tested whether the precision of the prediction is larger in the cases where these two tools agree. We here considered classifications only for the sequences where both tools had an identical PanHA classification. As a result, for 89% of those sequences ClassyFlu and IRD-CT agreed for the full HA segment classification. Thus this consensus approach improves accordance by 2% points compared to the results of IRD-CT alone (87%). As the classifications of one tool can agree on the full and PanHA sequence, although the classifications on the full sequence do not agree between tools, this is a conservative comparison to the accordance computed for a single method and the accuracy of predicted subtypes may be larger than 89% when IRD-CT and ClassyFlu agree.

### Subtype and H7 clade classification of newly emerging H7N9 viruses from China

In contrast to H5N1 HPAI viruses, no internationally approved cladistics for IAV of subtype H7 are available. Our tentative proposal is based on phylogenetic analysis by PhyML (see legend to Figure S1 for methodological details) of 198 full-length HA1 sequences of subtype H7 viruses selected from the Epiflu database. A total of 13 clades was distinguishable by high aLRT values (Figure S1). For each of these 13 H7 clades, a HMM was trained generatively.

As a sanity check, we tested how many of the same 198 H7 HA1 sequences were classified as H7. We repeated this test once for full length sequences and once for PanHA fragments. In both cases, all H7 sequences were correctly classified by ClassyFlu as H7. Also, to test a finer grained classification on the proposed clade level, we confirmed that all 17 sequences of the clade of H7N9 viruses that emerged in China early in 2013 (clade H7_China-1.2 in Figure S1) were reassigned to this clade, both when submitted to ClassyFlu as full length sequence and when submitted as PanHA fragment. In addition, all 23 H7N9/China sequences from GenBank and GISAID that were published between April 27th and July 18th, 2013, and that were not used to build the HMMs and the tree, were classified as clade H7_China-1.2. These test results can be considered additional anecdotal evidence for the accuracy of ClassyFlu. However given the lack of a larger number of recent independent H7 test sequences, above tests on H5 are more accurate and representative estimates of the performance.

## Discussion

ClassyFlu is able to very reliably and rapidly determine the subtype of a given full HA and NA sequence as demonstrated by its perfect classification on a test set of 870 HA and 98 NA sequences. On the harder task of assigning H5N1 clades to sequences it disagreed with the NCBI-annotated classification on 0.8% of the data, provided that the input sequences are full-length. These few cases were either minor disagreements or cases in which the WHO “clades” actually apear to be non-monophyletic.

The subtype and clade assignment analysis of a large set of very recent influenza A virus test sequences revealed a hierarchy in the accuracy of the classification methods. IRD-CT and ClassyFlu showed higher accordance compared to two semiautomated methods (BLAST, PHYLO) and provide the most consistent classification results. ClassyFlu is similarly accurate as IRD-CT and both are better than the ‘LAST’and ‘HYLO’ approaches. ClassyFlu is an accurate and simple alternative to IRD-CT. The advantages are not only high accuracy but also simplicity in handling. No database preparations are required and query sequences can simply be input in FASTA format. ClassyFlu always checks back on the same reference HMM database to assign sequences which prevents inconsistency in the analysis. Classification precision of IRD-CT relies on the structure and quality of the phylogenetic tree. The HMM database of ClassyFlu is based on the classified training sequences only and not on a particular tree. However, both tools use pre-classified sequences to classify user input sequences and their accuracy or usefulness may be affected by poorly chosen or erroneous input classifications. ClassyFlu can be easily trained for any other sequence-based assignment tasks where complex phylogenetic structures or swift diversifying evolution hamper easy classification by BLAST or rapid phylogenetic algorithms. We exemplified this by using HA sequences of the H7N9 avian influenza virus lineage which currently emerges in China and has caused a series of human infections. Both full length as well as PanHA fragment sequences of these viruses were promptly assigned to subtype H7. Provided international rules for H7 clade assignment will be proposed, ClassyFlu's profiles can easily be trained on these clades as well.

In conclusion, the newly developed influenza A virus HA sequence classification tool ClassyFlu, based on HMM profiling, provides highly accurate subtyping of full length HA sequences to subtypes and HPAIV H5N1 clades. A sequence fragment as short as 100 bp which spans the endoproteolytical cleavage site can be assigned with albeit reduced (86.3%) accuracy to H5N1 clades; however, accuracy is good enough for reliable HA and NA subtyping. Furthermore, the precision of the prediction is improved in the cases where ClassyFlu and IRD-CT agree (from 87% to 89%). Given the remaining error rate of just above 10% for classifying these short PanHA fragments into subclades of HPAIV H5N1, we recommend either to sequence longer fragments or to interpret predicted H5N1 clades based on panHA sequences as approximate.

## Supporting Information

Figure S1 A PhyML-directed phylogenetic analysis of subtype H7 HA sequences was based on the alignments of the open reading frames of the HA1 fragment (nucleotides 1–1023, representing amino acids 1–339) generated by with the aligment program MAFFT [12] and further optimized by manual editing using JalView [18]. The Akaike criterion calculated by JModeltest2 [19] was used to choose the most appropriate mutation model. PhyML was accessed via the ACTG server [20]. The resulting tree topology was visualized using FigTree (http://tree.bio.ed.ac.uk/software/figtree/). Further editing of the graphics was carried out with Inkscape (http://inkscape.org/).
(TIFF)

Table S1 Acknowledgement table for influenza sequences used that are in the GISAID database.
(XLS)
