# Supplementary figures and images for: ClassyFlu: Classification of Influenza A Viruses with Discriminatively Trained Profile-HMMs
Source: PLoS One. 2014 Jan 3;9(1):e84558. doi: 10.1371/journal.pone.0084558 (PMC3880301; doi:10.1371/journal.pone.0084558)

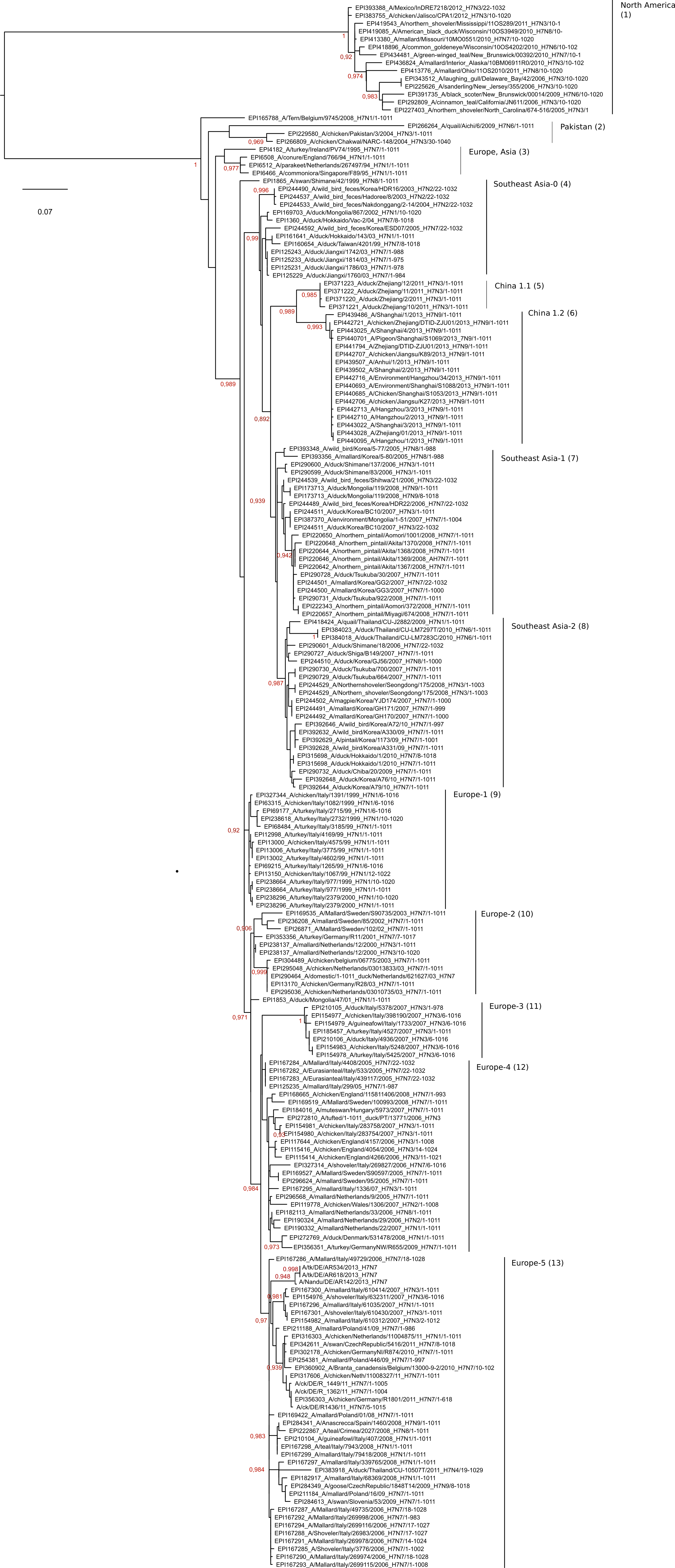

Supplement: Figure S1 — A PhyML-directed phylogenetic analysis of subtype H7 HA sequences was based on the alignments of the open reading frames of the HA1 fragment (nucleotides 1–1023, representing amino acids 1–339) generated by with the aligment program MAFFT [12] and further optimized by manual editing using JalView [18]. The Akaike criterion calculated by JModeltest2 [19] was used to choose the most appropriate mutation model. PhyML was accessed via the ACTG server [20]. The resulting tree topology was visualized using FigTree (http://tree.bio.ed.ac.uk/software/figtree/). Further editing of the graphics was carried out with Inkscape (http://inkscape.org/). (TIFF) [file pone.0084558.s001.tiff]
